# Does Alendronate reduce the risk of fracture in men? A meta-analysis incorporating prior knowledge of anti-fracture efficacy in women

**DOI:** 10.1186/1471-2474-6-39

**Published:** 2005-07-11

**Authors:** Anna M Sawka, Alexandra Papaioannou, Jonathan D Adachi, Amiram Gafni, David A Hanley, Lehana Thabane

**Affiliations:** 1 Department of Medicine, St. Joseph's Healthcare and McMaster University, Hamilton, Ontario, Canada; 2 Division of Endocrinology and Metabolism, McMaster University, Hamilton, Ontario, Canada; 3 Graduate Student in Health Research Methodology, McMaster University, Hamilton, Ontario, Canada; 4 Department of Medicine, Hamilton Health Sciences and McMaster University, Hamilton, Ontario, Canada; 5 Division of Geriatrics, Hamilton Health Sciences and McMaster University, Hamilton, Ontario, Canada; 6 Department of Clinical Epidemiology and Biostatistics, McMaster University, Hamilton, Ontario, Canada; 7 Division of Rheumatology, St. Joseph's Healthcare and McMaster University, Hamilton, Ontario, Canada; 8 Division of Endocrinology and Metabolism, Department of Medicine, University of Calgary, Calgary, Alberta, Canada; 9 Centre for Evaluation of Medicines, St. Joseph's Healthcare, Hamilton, Ontario, Canada

## Abstract

**Background:**

Alendronate has been found to reduce the risk of fractures in postmenopausal women as demonstrated in multiple randomized controlled trials enrolling thousands of women. Yet there is a paucity of such randomized controlled trials in osteoporotic men. Our objective was to systematically review the anti-fracture efficacy of alendronate in men with low bone mass or with a history of prevalent fracture(s) and incorporate prior knowledge of alendronate efficacy in women in the analysis.

**Methods:**

We examined randomized controlled trials in men comparing the anti-fracture efficacy of alendronate to placebo or calcium or vitamin D, or any combination of these. Studies of men with secondary causes of osteoporosis other than hypogonadism were excluded. We searched the following electronic databases (without language restrictions) for potentially relevant citations: Medline, Medline in Process (1966-May 24/2004), and Embase (1996–2004). We also contacted the manufacturer of the drug in search of other relevant trials. Two reviewers independently identified two trials (including 375 men), which met all inclusion criteria. Data were abstracted by one reviewer and checked by another. Results of the male trials were pooled using Bayesian random effects models, incorporating prior information of anti-fracture efficacy from meta-analyses of women.

**Results:**

The odds ratios of incident fractures in men (with 95% credibility intervals) with alendronate (10 mg daily) were: vertebral fractures, 0.44 (0.23, 0.83) and non-vertebral fractures, 0.60 (0.29, 1.44).

**Conclusion:**

In conclusion, alendronate decreases the risk of vertebral fractures in men at risk. There is currently insufficient evidence of a statistically significant reduction of non-vertebral fractures, but the paucity of trials in men limit the statistical power to detect such an effect.

## Background

Osteoporosis increases the risk of fragility fracture in both genders. In a population-based study of Canadians age 50 years by the Canadian Multicenter Osteoporosis Study Group, the prevalence of vertebral fractures was found to be 23.5% in men and 21.5% in women [1]. Alendronate, a potent oral bisphosphonate, decreases the risk of fractures in postmenopausal women with low bone mass or prevalent fractures, as established in a recent meta-analyses examining outcomes in thousands of women [2-4]. Less is known about the effect of alendronate in men, due to a paucity of randomized controlled trials. However, osteoporotic fractures are common in aging men; in fact the lifetime risk of a fracture of the spine, hip or distal radius is 13% for white men older than 50 years [5]. Our objective was to determine whether alendronate decreases risk of vertebral and non-vertebral fractures in men.

Upon initiating our review, we were aware of the paucity of trials examining anti-fracture efficacy of bisphosphonates in men. However, given that bisphosphonates decrease osteoclastic resorption in a mechanism independent of sex steroid status [6], we believed that the effect of alendronate in men would be similar to that previously observed in women. Therefore, the anti-fracture efficacy of alendronate in women would be relevant prior information to be incorporated in assessing treatment effects in men. Classical, frequentist, statistical methods do not offer the flexibility to incorporate relevant prior knowledge or beliefs in analysis of data, thus we sought an alternative statistical approach to analyze the results of our systematic review and we turned to Bayesian methods.

Bayesian statistical methods can explicitly and quantitatively incorporate relevant prior evidence in health technology assessment [7]. The foundation of Bayesian statistical methodology is Bayes' theorem, which is "a formula that shows how existing beliefs, formally expressed as probability distributions, are modified by new information" [8]. In Bayesian methodology, the conclusions of the analysis (known as the "posterior" inferences) are a result of modification of the "prior" data (in this case, known anti-fracture efficacy of alendronate in post-menopausal women), by new data collected (known as the "likelihood function", in this case, the data collected in men) [9]. Bayesian methodology is similar to clinical practice, as typically a clinician has a strong "prior" belief of, for example, a diagnosis such as osteoporosis prior to diagnostic testing, based on the clinical profile of the patient (such as age, gender, risk factors) and the results of diagnostic testing (analogous to a "likelihood function"), are used to confirm or refute those clinical suspicions and formulate a final conclusion (analogous to a "posterior" inference). Thus, Bayesian approaches are clinically intuitive. Moreover, results of Bayesian analyses are also more easily clinically translated than those of frequentist analyses [8]. For example, a Bayesian result tells us how likely is the result (such as an odds ratio), given the data [8]; as opposed to a frequentist result, which tell us how likely are the data given the null hypothesis. For these reasons, Bayesian methodology may be incorporated in healthcare medical decision-making [9]. Justification for the use of Bayesian approach in this study is the ability to directly answer the clinically relevant question: how likely is an osteoporotic man treated with alendronate to be protected from fracture given the current evidence in men and prior evidence in women? A classical frequentist analysis does not allow the flexibility to incorporate prior relevant information in the analysis and all relevant data from women would be ignored in such an analysis. Thus, a Bayesian approach was chosen as the primary analysis method for our study.

## Methods

### Design of the systematic review, inclusion and exclusion criteria

A systematic review of randomized-controlled trials was performed to determine the efficacy of alendronate (≥ 5 mg daily) in preventing vertebral and non-vertebral fractures in adult men with low bone mass or prevalent fracture(s). As a requirement for inclusion for each study, at least half the study population was required to consist of men, and the required minimum trial follow-up period was one year. A control group could be treated with calcium or vitamin D (any formulation, any dose), but no other active anti-osteoporotic therapies. In the case of duplicate publications on the same study population, only the study with the largest population size and longest follow-up period was included in our review. Studies were excluded if fracture outcome data were not provided, or if they included patients with bone or mineral disorders other than primary osteoporosis, with the exception of osteoporosis secondary to hypogonadism. Our focus was primarily on intention-to-treat data as such analyses are thought to avoid selection bias [10].

### Data sources and abstraction

One author searched the following electronic databases (without language restrictions): Medline, Medline-in-Process (1966-May 24/2004), Embase (1996–2004), and the Cochrane Central Register of Controlled Trials (1800 to May, 2004). The search terms used included the textwords "alendronate" or "fosamax", and the search was restricted to articles pertaining to adult male humans (age ≥ 19 years), clinical trials, controlled clinical trials, and randomized controlled trials (textwords "male" or "men" added in Embase) (Appendix). All citations were reviewed by two co-authors and citations which were deemed potentially relevant by either reviewer were examined in full by both investigators. Consensus was reached by both reviewers on all included studies. One co-author performed a PubMed search on the primary author of each included study to look for updated information on the included trials (such as updated follow-up data) or additional relevant trials and this search was restricted to studies including the text word "alendronate" or "fosamax" (up to May 24, 2004). The co-author who performed this secondary search examined the retrieved references and reviewed any potentially relevant trials with a second investigator. Consensus was again reached by both reviewers on whether any trial updates or new trials would be included. Merck-Frosst representatives at the Canadian and world-wide headquarters were contacted for information on any other potentially relevant trials not found by the electronic search. Male trial data were abstracted by one co-author and was checked by another. Fracture outcomes of individual trials in men were summarized using an intention-to-treat analysis principle. For randomized controlled trials meeting inclusion criteria in which fracture data were not published, primary authors were contacted by electronic mail for supplemental information.

### Statistical analyses and assumptions

The Bayesian approach was chosen because it allows formal incorporation of prior information in the analysis [7]. Bayesian random effects models predicting the log odds of vertebral fracture and non-vertebral fracture in alendronate-treated men incorporated prior information from published meta-analyses in postmenopausal women [2]. The effect of alendronate on fracture outcomes was assumed to be similar in both genders. In estimating the priors, we noted that in published random effects meta-analyses of postmenopausal women, the relative risk of vertebral fracture with alendronate therapy (≥ 5 mg daily) was 0.52 (95% confidence intervals [CI], 0.43, 0.65) (9360 women in eight studies); whereas the relative risk of non-vertebral fracture (alendronate ≥ 10 mg daily) was 0.51 (95% CI, 0.38, 0.69) (3723 women in six studies) [2]. For the purpose of our analyses, each relative risk was assumed to approach an odds ratio (OR), based on the assumption that relative risks and odds ratios are similar if the risk of the disease is very small, which was the case given the large sample sizes and low event rates. For each prior we assumed the normal distribution on log (OR) scale. Thus, the mean log OR was assumed to be -0.65 with variance 0.19 (precision 5.2) for vertebral fractures and -0.67 with variance 0.24 (precision 4.1) for non-vertebral fractures, respectively for alendronate-treated women. For the study heterogeneity, we assumed a Gamma prior distribution, determined on the basis that the mean heterogeneity between k studies is expected to be equal to the degrees of freedom, k-1 [21]. Thus, in calculating priors for study heterogeneity, we determined that a = 3.5 and b = 0.5 with k-1 = 7 for the analysis of vertebral fracture data; furthermore, a = 2.5 and b = 0.5 with k-1 = 5 for the analysis of non-vertebral fracture data.

WinBUGS version 1.4 (MRC Biostatistics Unit, Cambridge, UK) was used for all Bayesian statistical analyses. The prior knowledge of treatment effects of alendronate in women ("prior distribution") was incorporated in a hierarchical model and then the "likelihood function" of data in men was incorporated in this model for the same outcome. In order to transform the "prior" and "likelihood function" data to a "posterior" inference (final result), simulations were performed using Markov chain Monte Carlo Methods [9]. For each outcome, we performed 20,000 simulations, with Gibbs sampling of results for posterior distributions started at 2,500, after convergence was achieved in all models. A posterior distribution of the treatment effect of the results of alendronate on the log (OR) and inverse log (OR) scales was obtained for the outcomes of vertebral and non-vertebral fractures, respectively. The results were expressed as the posterior mean with corresponding 95% credibility interval [CRI]) on the inverse log (OR) scale (the latter interpreted as analogous to the OR). Using the traditional definition of 95% credibility interval, there is a 95% probability that the true treatment effect, in this case, the odds ratio, lies within this interval [20]. Comparison of the Bayesian results with the classical or frequentist random effects results using Review manager 4.1 (Cochrane Collaboration, Oxford, UK) was performed. The frequentist analysis did not incorporate any prior assumptions based on anti-fracture efficacy of alendronate in women and odds ratios of treatment effects were expressed as OR with the corresponding 95% confidence interval (CI).

## Results

### Results of search for relevant studies

**Figure 1:**
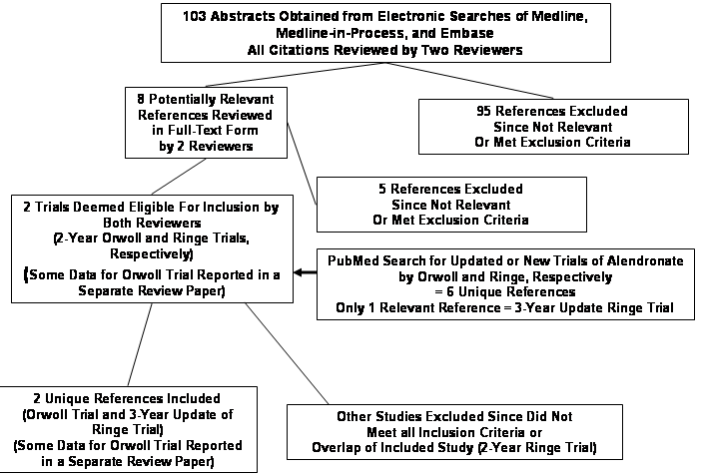
**Process of exclusion of studies. **The trials included at the final step are 1) Orwoll E et al. **Alendronate for the treatment of osteoporosis in men. ***N Engl J Med *2000;343(9):604-10; and 2) Ringe JD., et al. **Alendronate treatment of established primary osteoporosis in men: 3-year results of a prospective, comparative, two-arm study. ***Rheumatol Int *2004;24(2):110-3; with supplemental information on the Orwoll trial obtained from the review article, 3) Ringe JD, Orwoll E, et al. **Treatment of male osteoporosis: recent advances with alendronate. ***Osteoporos Int *2002; 13(3):195-9. The two-year analysis of the Ringe trial was excluded because of overlap of patients with the 3-year analysis: Ringe JD, et al. **Alendronate treatment of established primary osteoporosis in men: results of a 2-year prospective study. ***J Clin Endocrinol Metab *2001;86(11):5252–5255.

We obtained 103 unique references from our primary electronic searches of Medline/Medline-in-Process and Embase; no additional unique references were found using the Cochrane Central Register of Controlled Trials. No additional studies were revealed by Merck-Frosst. An additional six unique citations were examined upon searching under the name of the primary author of included trials. Nine of the references retrieved by our electronic searches were considered potentially relevant and reviewed in full-text form by two authors [11-19]. Only studies by Orwoll et al. [11] and Ringe et al. [12] (with information on numbers of patients with vertebral fractures from Orwoll documented in a subsequent paper [13]), met all inclusion criteria. The following studies, were excluded after review of full-text for the following reasons: Ringe 2002 (overlap of included study) [14], Gonnelli 2003 (lack of fracture outcomes, no additional information provided after attempting to contact primary author by electronic mail) [15], Finkelstein 2003 (active control of parathyroid hormone) [16], van der Poest 2002 (study of bone density changes in the setting of acute lower extremity fracture with immobilization) [17], Kushida 2002 (men comprised only 4% of the study population) [18], Ho 2000 (lack of control group) [19]. Details of the process of exclusion of trials are shown in Figure 1.

### Summary of alendronate trials in men

Characteristics of the included randomized controlled studies in men are shown in Table 1. Hypogonadal men were included in the Orwoll study [11] but not the Ringe study [12]. Approximately half of the men in each study had a history of one or more prevalent vertebral fractures before enrollment in the trial [11,12]. In the Orwoll study, an incident vertebral fracture was defined using the semi-quantitative method of Genant [22], as assessed by a radiologist blinded to treatment assignment at the University of California in San Francisco [11]. In the Ringe study, a vertebral fracture was defined by a new decrease in vertebral height of at least 20%, as assessed by a radiologist blinded to treatment assignment [12]. The Orwoll study was two years in duration [11], whereas the Ringe study extended for three years [12]. In both trials, the active treatment group received 10 mg of alendronate as well as 500 mg supplemental calcium orally daily [11,12]. In the Orwoll study, men in the treatment and control group received 400–450 IU vitamin D daily [11], whereas in the Ringe study, only men in the control arm received supplemental vitamin D in the form of 1 μg alfacalcidiol daily [12].

**Table 1:** Characteristics of included studies

**Study (reference)**	Sample (ALN*/ control) and duration	**Inclusion criteria**	**Age (years) (SD‡) Percentage prevalent vertebral fractures (VF, %)**	**Intervention/ Control**	**Blinding, randomization**	**Loss to follow-up or withdrew from study (n/N) (%)**
**Orwoll 2000 [11]**	146/95 (2 years)	Men with BMD† T-score = -2 at femoral neck and T-score = -1 at the lumbar spine; OR Men with T-score = -1 at the femoral neck and at least one vertebral or osteoporotic fracture	ALN: Mean age 63 (13) 49% VF Control: Mean age 63 (12) 52% VF	ALN*: 10 mg + 500 mg Calcium + 400–450 IU Vitamin D Control: 500 mg Calcium + 400–450 IU Vitamin D	- Double-blind - Radiologists reading vertebral x-rays blinded to intervention -Method of randomization unclear	38/241 (15.8%)
**Ringe 2004 [12]**	68/66 (3 years)	Men with BMD† T-score = -2.5 at femoral neck or lumbar spine, excluding hypogonadal men	ALN*: Mean age 52.7 (11.1) 54% VF Control: Mean age 53.3 (10.9) 53% VF	ALN*: 10 mg + 500 mg Calcium Control: 500 mg Calcium + 1 μg alfacalcidiol	- Open-label - Radiologists reading vertebral x-rays blinded to intervention -Method of randomization unclear	16/134 (11.9%)

*ALN, alendronate (daily dose)

\dagBMD, bone mineral density measurement by dual X-ray absorptiometry, compared to young adult male peak bone mass

\ddagSD, Standard deviation

In the Orwoll study, 4/146 alendronate-treated (2.7%) and 7/95 placebo-treated (7.4%) men sustained a vertebral fracture at two years [11,13]. In the Ringe study, 7/68 alendronate-treated men (10.3%) and 16/66 (24.2%) control participants sustained a vertebral fracture at three years [12]. Furthermore, In the Orwoll study, 6/146 alendronate-treated men (4.1%) and 5/95 men (5.3%) placebo-treated men sustained a non-vertebral fracture [11]. In the Ringe study, 6/68 men (8.8%) alendronate-treated men and 8/66 (12.1%) control participants sustained a non-vertebral fracture [12].

### Results of pooled analyses

**Figure 2:**
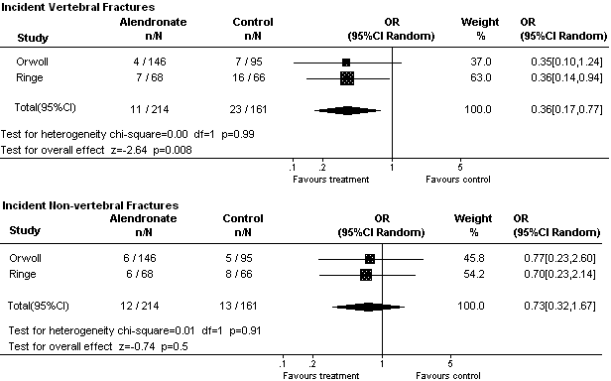
**Pooled random effects meta-analyses of vertebral and non-vertebral fracture outcomes with alendronate treatment (frequentist models). **OR, Odds Ratio; 95% CI Random, 95% confidence interval using a frequentist random effects model for pooling the data from studies

Upon incorporating the prior information from postmenopausal women with the male data in the Bayesian random effects model, the OR of vertebral fracture in alendronate-treated men was 0.44 (95% CRI 0.23, 0.83) and the OR of non-vertebral fracture was 0.60 (95% CRI 0.29, 1.44). Similar results were found using the frequentist random effects models of male data (vertebral fracture OR 0.36, 95% CI 0.17, 0.77; non-vertebral fracture OR 0.73, 95% CI, 0.32, 1.67), without incorporation of any of the data from women (Figure 2).

## Discussion

Limitations of our systematic review include the paucity of trial data from men, the small sample sizes in trials, the variations in trial duration between studies, and the inconsistency of calcium and vitamin D formulations between trials (with the possibility that the alfacalcidiol used in the Ringe study could be considered a form of active therapy [23]). We were also unable to perform a per protocol sensitivity analysis of the anti-fracture efficacy of alendronate in men who were compliant with therapy as these data were not published in the primary trials. We did not include unpublished data. We also did not contact author to clarify details of the randomization procedures. Merits of our study include its systematic nature, including both Bayesian and traditional frequentist analyses of available data, and the examination of clinically relevant fracture outcomes. Of note, the numerical estimates of odds ratios and their respective credibility or confidence intervals were similar using Bayesian and frequentist analyses in this study. These findings are not surprising, given that the treatment effects of alendronate in women, from whom prior information was derived, were similar to those observed in men. The precision of our estimate was however slightly improved using a Bayesian approach for the outcome of non-vertebral fractures as seen by the slightly narrower credibility interval than confidence interval for that outcome. Credibility intervals can be narrower using a Bayesian approach than confidence intervals obtained using a frequentist approach because of additional data provided by the priors [9].

Given that the results of our Bayesian and frequentist meta-analyses were similar, was there any advantage to the Bayesian approach? The basic question posed using a frequentist approach is how likely are the data given the null hypothesis? In contrast, the question posed from a Bayesian perspective is, how likely is the odds ratio, given the data [8]? Furthermore, the interpretation of a traditional 95% confidence interval is that given a long series of such intervals, 95% of them should contain the true value of the odds ratio [9]. In contrast, in interpreting a 95% credibility interval, there is a 95% probability that the true value of the parameter lies within this interval [9]. Thus, the clinical question posed and the interpretation of the probability interval, are more clinically intuitive from a Bayesian perspective. Moreover, the Bayesian approach allowed the flexibility to incorporate clinically relevant evidence from trials in women in this meta-analysis of alendronate treatment in men.

In the future, more trials of active osteoporotic therapies and their effects on fracture outcomes need to be performed in both genders.

## Conclusion

We conclude that alendronate (10 mg daily) decreases the risk of vertebral fractures in men with low bone mass or fractures, but there is currently insufficient evidence to prove a significant effect on non-vertebral fractures in this population. The paucity of osteoporosis therapy trials in men as well as the relative infrequency of non-vertebral fractures in the control groups may have restricted our statistical power to detect a significant reduction of non-vertebral fractures. Of note, the relative risk of vertebral fracture in alendronate-treated men was similar to that previously observed in a meta-analysis of data from postmenopausal women [2].

## Appendix

### Electronic search strategy for potentially relevant studies

For Medline and Medline-in-Process (1966 to May 24, 2004), Cochrane Central Register of Controlled Trials (1800 to May 24, 2004): "alendronate" or "fosamax" - restricted to adult male humans (age ≥ 19 years), clinical trials, controlled clinical trials, and randomized controlled trials.

For Embase 1996 to May 24, 2004: "alendronate" or "fosamax" AND "male" or "men"- restricted to adult male humans (age ≥ 19 years), clinical trials, controlled clinical trials, and randomized controlled trials.

For PubMed (up to May 24, 2004): name and initials of authors of included studies AND "alendronate" or "fosamax"

## Competing interests

Dr. Sawka is a Skeletal Health Scholar funded, in part, by the Canadian Institutes of Health Research. Dr. Sawka is also a Fellow in Health Economics at McMaster University, partly funded by an unrestricted educational grant from Hoffmann-La Roche.

Dr. Papaioannou's competing interests include: Aventis, Eli Lilly, Merck, Novartis, and Procter and Gamble.

Dr. Adachi, Consultant to: Amgen, Astra Zeneca, Aventis, Eli Lilly, Glaxo Smith Kline, Merck, Novartis, Procter and Gamble, and Hoffman-La Roche.

Dr. Hanley's competing interests include consultancies with, honoraria for speaking from, or involvement in research with, the following companies or organizations : Amgen, Astra-Zeneca, Aventis, the Dairy Farmers of Canada, Eli Lilly, Merck, Novartis, NPS Pharmaceuticals, Pfizer, Procter and Gamble, Hoffman-La Roche, and Wyeth.

The other co-authors have no competing interests to declare.

## Authors' contributions

All co-authors reviewed the manuscript and made suggestions for revisions. The project idea was conceived by Dr. Sawka. Analyses were performed by Dr. Sawka, with input from Dr. Thabane. The manuscript was written and revised by Dr. Sawka.

## Pre-publication history

The pre-publication history for this paper can be accessed here:


